# Supplementary material for: Patient Preferences for Long-Term Implant Care in Cochlear, Glaucoma and Cardiovascular Diseases
Source: Int J Environ Res Public Health. 2023 Jul 13;20(14):6358. doi: 10.3390/ijerph20146358 (PMC10378795; doi:10.3390/ijerph20146358)
Supplement: Supplementary file 1 [file ijerph-20-06358-s001.zip › ijerph-2437412-supplementary.pdf]

**Patient preferences for long-term implant care – results from discrete choice experiments in the context of cochlear, glaucoma and cardiovascular implants**

Copy of CI-DCE survey (exemplary) – German version (orig.) and English version (translated)

*Note from the author:* CI-DCE were conducted after GI- and CVI-DCE which is why feedback we received from CI-experts relating to the DCE (i.e. more detailed task instructions) could not be applied to GI- and CVI-DCE. Therefore, the instructions of the DCEs vary between implant areas. However, participants were able to leave comments at the end of each DCE and the participants' answers did not indicate any problems in understanding the task in the GI- and CVI-DCE.

## Survey A - Cochlea Implants (CI)

*1. German Version (original)*

### Instruktionen

### Erklärung der Aufgabe

Nun beginnen wir mit den Discrete-Choice-Experimenten bzw. den Auswahl-Aufgaben.

Stellen Sie sich bei der Aufgabenbearbeitung bitte folgendes Szenario vor: Sie haben sich für die Versorgung mit einem (neuen) Cochlea-Implantat (CI) entschieden. Dafür stehen Ihnen zwei Versorgungspakete zur Verfügung. Welche der dargestellten Optionen bevorzugen Sie?

Ihnen werden insgesamt **18 Auswahl-Aufgaben** präsentiert. Jede Aufgabe enthält zwei Versorgungspakete (Paket A und Paket B). Die Pakete werden in jeder Aufgabe mit **denselben 6 Merkmalen (linke Spalte)** beschrieben, aber die **Merkmalsausprägungen (rechte Spalten) ÄNDERN** sich. Schauen Sie sich also bitte die Ausprägungen genau an und entscheiden, welches Versorgungspaket Sie bevorzugen.

### BEISPIEL-Darstellung

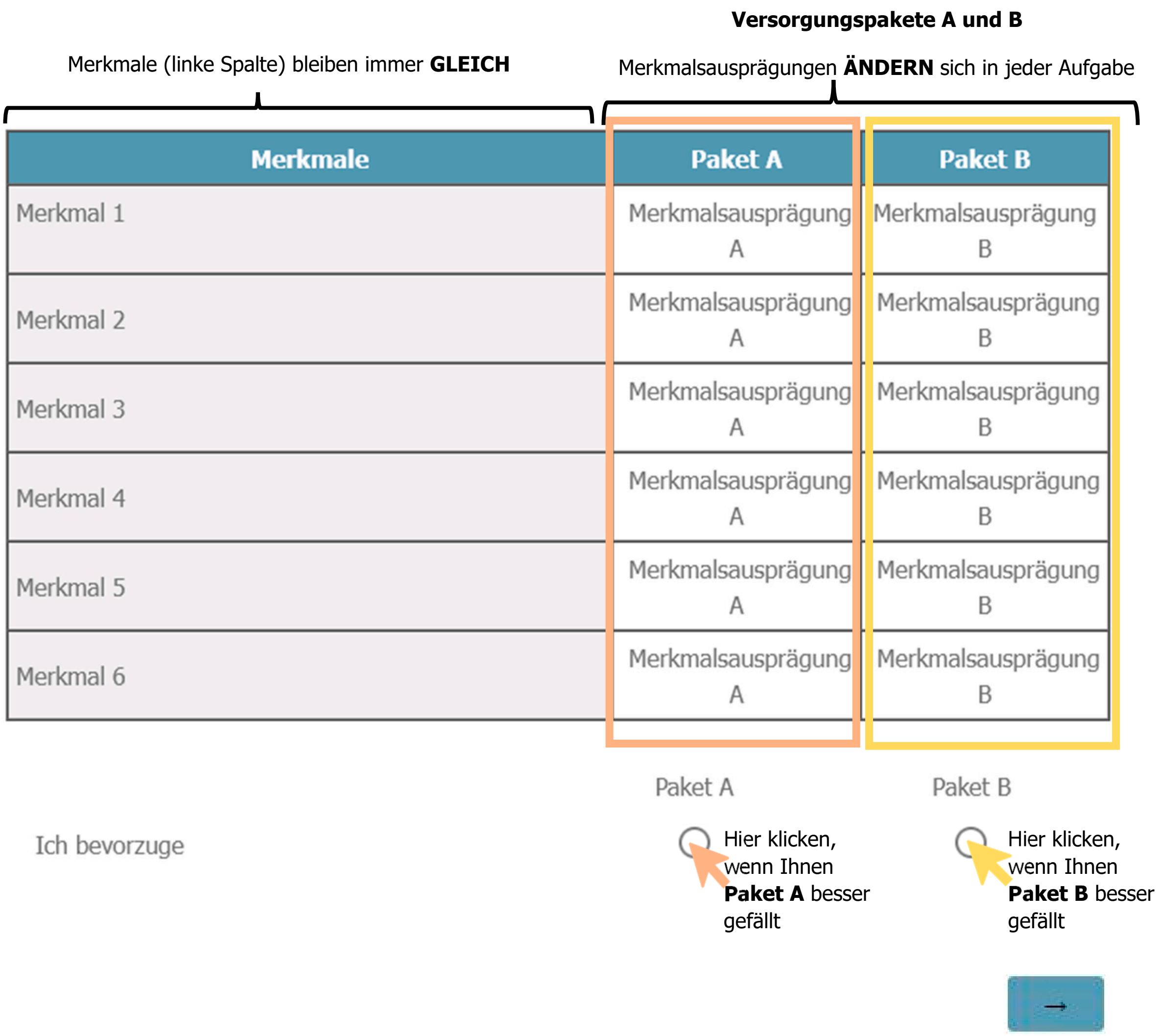

Nachdem Sie ihr bevorzugtes Paket ausgewählt haben, klicken Sie auf die **Pfeil-Taste**. Damit wird Ihre Antwort erfasst und eine neue Aufgabe mit **veränderten Merkmalsausprägungen der Pakete A und B** erscheint. Bitte entscheiden Sie nun erneut, welches Paket Sie bevorzugen. Insgesamt bearbeiten Sie auf diese Weise **18 Aufgaben**.

Hinweis: Bitte wählen Sie immer dasjenige Paket aus, welches Ihren eigenen Vorlieben am nächsten kommt - auch wenn Sie bei einer "echten" Entscheidung möglicherweise keines der Pakete gewählt hätten. Denn es geht hierbei nicht darum, wie Sie die Versorgung in der Realität erleben bzw. erlebt haben, sondern wie Sie sich eine Implantat-Versorgung eher wünschen würden, wenn Ihnen genau diese beiden Optionen angeboten würden. Die präsentierten Pakete und Fragen sind also hypothetisch, d.h. nur im Rahmen der hier durchgeführten gedanklichen Experimente werden Sie vor die Wahl zwischen diesen Versorgungspaketen gestellt. **Ihre Entscheidung hat keinen Einfluss auf Ihre tatsächliche aktuelle Behandlung.**

In dieser Studie geht es um Versorgungsfragen im Bereich von Cochlea-Implantaten (CI). Sind Sie eine Trägerin bzw. ein Träger eines Cochlea-Implantats (CI) oder haben eine Indikation für ein solches Implantat?

- ☐ Ja
- ☐ Nein

---

**Auswahlaufgabe (Block 1) [exemplarisch]**

Stellen Sie sich vor, Ihnen stehen die folgenden Versorgungspakete A und B in Bezug auf die Versorgung mit einem Cochlea-Implantat (CI) zur Verfügung.

Zur Erinnerung: Die linke Spalte "Merkmale" bleibt immer gleich, aber die Angaben zu **Paket A und Paket B wurden verändert**. Bitte schauen Sie sich die Pakete A und B deswegen **genau** an.

Welches Paket bevorzugen Sie?

| Merkmale | Paket A | Paket B |
|---|---|---|
| Finale Entscheidung über Implantation des CI-Modells eines bestimmten Herstellers liegt... | alleinig bei Ihrer/m Ärztin/Arzt | bei Ihnen und Ihrer/m Ärztin/Arzt |
| Erhalt von Informationen über (Weiter-) Entwicklungen in der Versorgung des CI: | automatisch vom Hersteller | durch eigene Recherche |
| Andere Behandlungsmöglichkeiten nach CI-Implantation, welche noch erforscht werden und eventuell in Zukunft zur Verfügung stehen… | werden ausgeschlossen | bleiben erhalten |
| Kompatibilität mit neueren CI-Modellen und Zubehör oder Geräten anderer Hersteller: | kompatibel | nicht kompatibel |

| | | |
|---|---|---|
| Durchführung der Nachsorge: | nach fachlichem Bedarf wechselndes Personal | fester Stab an in Austausch stehendem Personal |
| Die Aufklärung hinsichtlich Anpassungen, Entscheidungen und Neuerungen bezüglich des CI... | sehr ausführlich | wenig ausführlich |
| Ich bevorzuge | Paket A<br>O | Paket B<br>O |

**Soziodemographischer Fragebogen**

Vielen Dank für die Bearbeitung der Präferenzwahl-Aufgabe.

Zum Schluss möchten wir Sie bitten, ein paar Angaben zu Ihrer Person zu machen.

Zur Erinnerung: Die Daten werden vollständig anonymisiert gespeichert, verarbeitet und analysiert. Es werden keine identitätsbezogenen Daten wie Namen oder Kontaktdaten erfragt. Zu keinem Zeitpunkt der Studie werden Rückschlüsse auf Ihre Identität gemacht.

Wie alt sind Sie?

| |
|---|
| |

Mit welchem Geschlecht identifizieren Sie sich?

- ☐ weiblich
- ☐ männlich
- ☐ divers

Gibt es Ihrerseits noch Anmerkungen zur Durchführung dieser Studie, die Sie uns gerne mitteilen möchten?

- ☐ Nein
- ☐ Ja, und zwar:

| |
|---|
| |

## 2. *English Version (translated)*

### Instructions

### Explanation of the task

We will now start with the discrete choice experiments resp. the choice tasks.

Please imagine the following scenario while working on the task: You have decided to receive a (new) cochlear implant (CI). Two fitting packages are available to you. Which of the options presented do you prefer?

You will be presented with a total of **18 choice tasks**. Each task contains two fitting packages (Package A and Package B). The packages are described with **the same 6 attributes (left column)** in each task, but the **attribute specifications (right columns) CHANGE**. So please look at the attributes carefully and decide which care package you prefer.

### EXAMPLE display

**Care option A and B**

Attribute specifications **CHANGE** in each task

Attributes (left column) always remain **SAME**

| Attributes | Care option A | Care option B |
|---|---|---|
| Attribute 1 | Attribute specification A | Attribute specification B |
| Attribute 2 | Attribute specification A | Attribute specification B |
| Attribute 3 | Attribute specification A | Attribute specification B |
| Attribute 4 | Attribute specification A | Attribute specification B |
| Attribute 5 | Attribute specification A | Attribute specification B |
| Attribute 6 | Attribute specification A | Attribute specification B |

| I prefer | Care option A | Care option B |
|---|---|---|
| | Click here if you prefer **option A** | Click here if you prefer **option B** |

→

After you have chosen your preferred package, click on the **arrow button**. This will register your response and a new task with **changed attribute specifications of packages A and B** will appear. Please decide again which package you prefer. In total, you will complete **18 tasks** in this way.

Note: Please always choose the package that comes closest to your own preferences - even though you may not have chosen any of the packages if you had made a "real" decision. This is because it is not about how you experience or have experienced care in reality, but how you would prefer implant care if you were offered exactly these two options. The packages and questions presented are therefore hypothetical, i.e. only within the framework of the mental experiments carried out here are you confronted with the choice between these care packages. **Your decision has no influence on your actual current treatment.**

This study is about cochlea implant care. Are you a cochlear implant wearer or do you have an indication for such an implant?

- ☐ Yes
- ☐ No

---

**Choice Task [exemplary]**

Imagine that the following care packages A and B are available to you in relation to the provision of a cochlear implant (CI).

*As a reminder:* The left column " Attributes" always remains the same, but the specifications for **package A and package B have been changed**. Please take a **close look** at packages A and B for this reason.

Which package do you prefer?

| Attributes | Care option A | Care option B |
|---|---|---|
| Final decision on the implantation of a specific manufacturer's CI model lies... | Only with your doctor | With you and your doctor |
| Obtaining information about developments regarding the CI and care context: | Automatically by manufacturer | Through own research |
| Other treatment options after CI implantation, which are still being researched and may be available in the future... | Are excluded | Remain available |
| Compatibility with newer CI models and accessories or devices from other manufacturers | Compatible | Not compatible |
| Carrying out aftercare: | Varying professionals according to specific needs | Fixed staff of professionals who are in exchange |
| Education regarding adjustments, decisions and innovations regarding your CI is... | Very comprehensive | Little comprehensive |

| | Care option A | Care option B |
|---|---|---|
| I prefer | O | O |

**Socio-demographic questionnaire**

Thank you for completing the preference choice task.

Finally, we would like to ask you to provide a few details about yourself.

As a reminder, the data will be stored, processed and analysed completely anonymously. No identity-related data such as names or contact details will be requested. At no point in the study will any conclusions be drawn about your identity.

How old are you?

Which gender do you identify with?

- ☐ female
- ☐ male
- ☐ diverse

Are there any further comments on your part about the conduct of this study that you would like to share with us?

- ☐ No
- ☐ Yes:

## Survey B - Glaucoma Implants (GI)

1. *German Version (original)*

### Instruktionen

### Erklärung der Aufgabe

Nun beginnen wir mit den Discrete-Choice-Experimenten bzw. der Präferenzwahl-Aufgabe.

Stellen Sie sich bei der Aufgabenbearbeitung bitte folgendes Szenario vor: Sie haben sich für die Versorgung mit einem Glaukom-Implantat (GI) entschieden. Dafür stehen Ihnen verschiedene Angebote bzw. Versorgungspakete zur Verfügung. Es soll darum gehen, welches der dargestellten Pakete Sie bevorzugen.

Ihnen werden insgesamt **18 Szenarien** präsentiert. Jedes Szenario enthält zwei Versorgungspakete (Paket A und Paket B). Die Pakete werden in jedem Szenario mit denselben 6 Merkmalen beschrieben, aber die Merkmalsausprägungen unterscheiden sich. Schauen Sie sich also bitte die Ausprägungen genau an und entscheiden, welches Versorgungspaket Sie bevorzugen.

Bitte wählen Sie immer dasjenige Paket aus, welches Ihren eigenen Vorlieben am nächsten kommt - auch wenn Sie bei einer "echten" Entscheidung möglicherweise keines der Pakete gewählt hätten. Denn es geht hierbei nicht darum, wie Sie die Versorgung in der Realität erleben bzw. erlebt haben, sondern wie Sie sich eine Implantat-Versorgung eher wünschen würden, wenn Ihnen genau diese beiden Optionen angeboten würden. Die präsentierten Pakete und Fragen sind also hypothetisch, d.h. nur im Rahmen der hier durchgeführten gedanklichen Experimente werden Sie vor die Wahl zwischen diesen Versorgungspaketen gestellt. **Ihre Entscheidung hat keinen Einfluss auf Ihre tatsächliche aktuelle Behandlung.**

In dieser Studie geht es um die Präferenzwahl im Bereich von Glaukom-Implantaten (GI). Sind Sie ein Träger bzw. eine Trägerin eines Glaukom-Implantats (z.B Stent, Drainageröhrchen, MicroShunt, Ventil-Implantat) oder kommt ein solches Implantat für Sie in Frage?

- ☐ Ja
- ☐ Nein

### Auswahlaufgabe (Block 1) [exemplarisch]

Stellen Sie sich vor, Ihnen stehen die folgenden Versorgungspakete A und B in Bezug auf die Versorgung mit einem Glaukom-Implantat (GI) zur Verfügung. Bitte schauen Sie sich die Pakete **genau** an. Welches Paket bevorzugen Sie?

| | Paket A | Paket B |
|---|---|---|
| Finale Entscheidung über Behandlungsoptionen inkl. Implantat-Modell liegt... | alleinig bei Ihrer/m Ärztin/Arzt | bei Ihnen und Ihrer/m Ärztin/Arzt |
| Mögliche Maßnahmen für den Fall, dass das Implantat nicht (mehr) funktioniert: | Verbleib im Auge | Nachbesserung mithilfe eines Eingriffs |

| | | |
|---|---|---|
| Erfolgschancen, dass 2 Jahre nach Implantation weiterhin keine Glaukom-Medikamente benötigt werden, liegen bei... | mehr als 50% | mehr als 75% |
| Informationen, die über Implantation und Nachsorge hinausgehen (z.B zu Ernährung, Tropfen gegen Augentrockenheit, Brille etc.) erhalten Sie... | im Rahmen der Implantat-Versorgung von ärtztlicher Seite | von unabhängigen Informationsstellen (z.B. Glaukomforum) |
| Statistiken und Erfahrungswerte zur Implantation in der behandelnden Klinik... | sind nicht verfügbar | sind verfügbar |
| Informationsaustausch zwischen verschiedenen Behandler:innen (z.B. niedergelassener Augenarzt, Hausärztin und Klinik)... | koordinieren Sie selbst | findet automatisch statt |
| Ich bevorzuge | Paket A<br>O | Paket B<br>O |

**Soziodemographischer Fragebogen**

Vielen Dank für die Bearbeitung der Präferenzwahl-Aufgabe.

Zum Schluss möchten wir Sie bitten, ein paar Angaben zu Ihrer Person zu machen.

Zur Erinnerung: Die Daten werden vollständig anonymisiert gespeichert, verarbeitet und analysiert. Es werden keine identitätsbezogenen Daten wie Namen oder Kontaktdaten erfragt. Zu keinem Zeitpunkt der Studie werden Rückschlüsse auf Ihre Identität gemacht.

Wie alt sind Sie?

Mit welchem Geschlecht identifizieren Sie sich?

- ☐ weiblich
- ☐ männlich
- ☐ divers

Gibt es Ihrerseits noch Anmerkungen zur Durchführung dieser Studie, die Sie uns gerne mitteilen möchten?

- ☐ Nein
- ☐ Ja, und zwar:

*2. English Version (translated)*

**Instructions**

**Explanation of the task**

Now we will start with the discrete choice experiments or the preference choice task.

As you work through the task, please imagine the following scenario: You have decided to receive a glaucoma implant (GI). There are various care options available to you. The task is to decide which of the presented options you prefer.

You will be presented with a total of **18 scenarios**. Each scenario contains two care options (options A and option B). The care options are described with the same 6 characteristics in each scenario, but the option's characteristics differ. So please take a close look at the characteristics and decide which care option you prefer.

Please always choose the option that comes closest to your own preferences - even though you might not have chosen any of the options in a "real" decision. This is because it is not about how you experience or have experienced the glaucoma care in reality, but what option you would prefer if you were offered exactly these two options. The care options and questions presented are hypothetical, i.e. only within the framework of the mental experiments in this study are you confronted with the choice between these care options. **Your decision has no influence on your actual current treatment**.

This study examines preferences in the area of glaucoma implants (GI). Are you a glaucoma implant wearer (e.g. stent, drainage tube, MicroShunt, valve implant) or is such an implant an option for you?

- ☐ Yes
- ☐ No

**Choice Task [exemplary]**

Imagine that the following care options A and B are available to you in relation to the provision of a glaucoma implant (GI). Please look **closely** at the care options. Which care option do you prefer?

| Attributes | Care option A | Care option B |
|---|---|---|
| Final decision on the implantation of a specific manufacturer's CI model lies... | Only with your doctor | With you and your doctor |
| Corrective measure in the event that the implant does not (or no longer) work | Automatically by manufacturer | Through own research |
| Chances of success of still not needing glaucoma medication 2 years after implantation are: | Over 50% | Over 75% |

| | | |
|---|---|---|
| Information that goes beyond implantation and aftercare (e.g., on nutrition, drops for dry eyes, glasses, etc.) will be provided… | In the context of implant care from the medical side | From independent information sources (e.g., glaucoma forum) |
| Statistics and empirical values for implantation in the treating clinic… | Are not available | Are available |
| Exchange of information between different health professionals (e.g., resident ophthalmologist, family doctor and clinic) | You coordinate yourself | Takes place automatically |
| I prefer | Care option A<br>O | Care option B<br>O |

**Socio-demographic questionnaire**

Thank you for completing the preference choice task.

Finally, we would like to ask you to provide a few details about yourself.

As a reminder, the data will be stored, processed and analysed completely anonymously. No identity-related data such as names or contact details will be requested. At no point in the study will any conclusions be drawn about your identity.

How old are you?

Which gender do you identify with?

- ☐ female
- ☐ male
- ☐ diverse

Are there any further comments on your part about the conduct of this study that you would like to share with us?

- ☐ No
- ☐ Yes:

## Survey C - Cardiovascular Implants (CVI)

1. *German Version (original)*

**Instruktionen**

### Erklärung der Aufgabe

Nun beginnen wir mit den Discrete-Choice-Experimenten bzw. der Präferenzwahl-Aufgabe.

Stellen Sie sich bei der Aufgabenbearbeitung bitte folgendes Szenario vor: Sie haben sich für die Versorgung mit einem kardiovaskulären Implantat entschieden. Dafür stehen Ihnen verschiedene Angebote bzw. Versorgungspakete zur Verfügung. Es soll darum gehen, welches der dargestellten Pakete Sie bevorzugen.

Ihnen werden insgesamt **12 Szenarien** präsentiert. Jedes Szenario enthält zwei Versorgungspakete (Paket A und Paket B). Die Pakete werden in jedem Szenario mit denselben 6 Merkmalen beschrieben, aber die Merkmalsausprägungen unterscheiden sich. Schauen Sie sich also bitte die Ausprägungen genau an und entscheiden, welches Versorgungspaket Sie bevorzugen.

Bitte wählen Sie immer dasjenige Paket aus, welches Ihren eigenen Vorlieben am nächsten kommt - auch wenn Sie bei einer "echten" Entscheidung möglicherweise keines der Pakete gewählt hätten. Denn es geht hierbei nicht darum, wie Sie die Versorgung in der Realität erleben bzw. erlebt haben, sondern wie Sie sich eine Implantat-Versorgung eher wünschen würden, wenn Ihnen genau diese beiden Optionen angeboten würden. Die präsentierten Pakete und Fragen sind also hypothetisch, d.h. nur im Rahmen der hier durchgeführten gedanklichen Experimente werden Sie vor die Wahl zwischen diesen Versorgungspaketen gestellt. **Ihre Entscheidung hat keinen Einfluss auf Ihre tatsächliche aktuelle Behandlung.**

In dieser Studie geht es um die Präferenzwahl im Bereich von kardiovaskulären Implantaten (KI). Sind Sie ein Träger bzw. eine Trägerin kardiovaskulären Implantats oder kommt ein solches Implantat für Sie in Frage?

- ☐ Ja
- ☐ Nein

### Auswahlaufgabe (Block 1) [exemplarisch]

Stellen Sie sich vor, Ihnen stehen die folgenden Versorgungspakete A und B in Bezug auf die Versorgung mit einem Glaukom-Implantat (GI) zur Verfügung. Bitte schauen Sie sich die Pakete **genau** an. Welches Paket bevorzugen Sie?

| | Paket A | Paket B |
|---|---|---|
| Finale Entscheidung über Behandlungsoptionen inkl. Implantat-Art liegt... | alleinig bei Ihnen | alleinig beim ärztlichen Team |

| | | |
|---|---|---|
| Informationen, die über Implantation und Nachsorge hinausgehen (z.B. zu Ernährung, Lebensstil etc.) erhalten Sie... | im Rahmen der Implantat-Versorgung von ärztlicher Seite | von unabhängigen Informationsstellen (z.B Dt. Herzstiftung) |
| Statistiken und Erfahrungswerte zum Einsatz von kardiovaskulären Implantaten im behandelnden Herzzentrum... | sind nicht verfügbar | sind verfügbar |
| Informationsaustausch zwischen verschiedenen Behandler:innen (z.B. niedergelassener Hausarzt, Herzzentrum und Ärztinnen anderer Fachrichtungen)... | koordinieren Sie selbst | findet automatisch statt |
| Ein langfristiger Erfolg der Behandlung kann gesichert werden durch... | lebenslange Medikamenteneinnahme (z.B. Blutverdünner) | neues Implantat jede 10 Jahre |
| Einsatz des Implantats erfolgt... | minimalinvasiv (ohne Brustkorböffnung) | chirurgisch (Brustkorböffnung) |
| | Paket A | Paket B |
| Ich bevorzuge | O | O |

---

**Soziodemographischer Fragebogen**

Vielen Dank für die Bearbeitung der Präferenzwahl-Aufgabe.

Zum Schluss möchten wir Sie bitten, ein paar Angaben zu Ihrer Person zu machen.

Zur Erinnerung: Die Daten werden vollständig anonymisiert gespeichert, verarbeitet und analysiert. Es werden keine identitätsbezogenen Daten wie Namen oder Kontaktdaten erfragt. Zu keinem Zeitpunkt der Studie werden Rückschlüsse auf Ihre Identität gemacht.

Wie alt sind Sie?

| |
|---|
| |

Mit welchem Geschlecht identifizieren Sie sich?

- ☐ weiblich
- ☐ männlich
- ☐ divers

Gibt es Ihrerseits noch Anmerkungen zur Durchführung dieser Studie, die Sie uns gerne mitteilen möchten?

- ☐ Nein
- ☐ Ja, und zwar:

*2. English Version (translated)*

**Instructions**

**Explanation of the task**

Now we will start with the discrete choice experiments or the preference choice task.

As you work through the task, please imagine the following scenario: You have decided to receive a cardiovascular implant (CVI). There are various care options available to you. The task is to decide which of the presented options you prefer.

You will be presented with a total of **12 scenarios**. Each scenario contains two care options (options A and option B). The care options are described with the same 6 characteristics in each scenario, but the option's characteristics differ. So please take a close look at the characteristics and decide which care option you prefer.

Please always choose the option that comes closest to your own preferences - even though you might not have chosen any of the options in a "real" decision. This is because it is not about how you experience or have experienced the CVI care in reality, but what option you would prefer if you were offered exactly these two options. The care options and questions presented are hypothetical, i.e. only within the framework of the mental experiments in this study are you confronted with the choice between these care options. **Your decision has no influence on your actual current treatment**.

This study examines preferences in the area of cardiovascular implants (CVI). Are you a cardiovascular implant wearer or is such an implant an option for you?

☐ Yes
☐ No

**Choice Task [exemplary]**

Imagine that the following care options A and B are available to you in relation to the provision of cardiovascular implant (CVI). Please look **closely** at the care options. Which care option do you prefer?

| Attributes | Care option A | Care option B |
|---|---|---|
| Final decision on the implantation of a specific manufacturer's CI model lies... | Only with you | Only with your doctors |
| Information that goes beyond implantation and aftercare (e.g., on nutrition, lifestyle, etc.) will be provided… | In the context of implant care from the medical side | From independent information sources (e.g., German Heart Foundation) |
| Statistics and empirical values for stent implantation in the treating heart clinic… | Are not available | Are available |

| | | |
|---|---|---|
| Exchange of information between different health professionals (e.g., family doctor, clinic and other health professionals) | You coordinate yourself | Takes place automatically |
| A long-term success of the treatment can be secured by… | Lifelong medication (e.g., blood thinners) | New implant every 10 years |
| The implant is inserted… | Minimally invasive (without opening the chest) | Surgical (chest opening) |
| | Care option A | Care option B |
| I prefer | O | O |

**Socio-demographic questionnaire**

Thank you for completing the preference choice task.

Finally, we would like to ask you to provide a few details about yourself.

As a reminder, the data will be stored, processed and analysed completely anonymously. No identity-related data such as names or contact details will be requested. At no point in the study will any conclusions be drawn about your identity.

How old are you?

Which gender do you identify with?

- ☐ female
- ☐ male
- ☐ diverse

Are there any further comments on your part about the conduct of this study that you would like to share with us?

- ☐ No
- ☐ Yes:
